# Biotransformation, Pharmacokinetics, and Pharmacological Activities of Ginsenoside Rd Against Multiple Diseases

**DOI:** 10.3389/fphar.2022.909363

**Published:** 2022-07-19

**Authors:** Jing Li, Qingxia Huang, Yao Yao, Peng Ji, E. Mingyao, Jinjin Chen, Zepeng Zhang, Hongyu Qi, Jiaqi Liu, Zhaoqiang Chen, Daqing Zhao, Lei Zhou, Xiangyan Li

**Affiliations:** ^1^ Jilin Ginseng Academy, Key Laboratory of Active Substances and Biological Mechanisms of Ginseng Efficacy, Ministry of Education, Jilin Provincial Key Laboratory of Bio-Macromolecules of Chinese Medicine, Changchun University of Chinese Medicine, Changchun, China; ^2^ Research Center of Traditional Chinese Medicine, College of Traditional Chinese Medicine, Changchun University of Chinese Medicine, Changchun, China; ^3^ College of Acupuncture and Tuina, Changchun University of Chinese Medicine, Changchun, China; ^4^ Department of Pathology, Affiliated Hospital of Changchun University of Chinese Medicine, Changchun, China

**Keywords:** *Panax ginseng* C.A. Mey., ginsenoside Rd, biotransformation, pharmacokinetics, molecular mechanisms

## Abstract

*Panax ginseng* C.A. Mey. has a history of more than 4000 years and is widely used in Asian countries. Modern pharmacological studies have proved that ginsenosides and their compounds have a variety of significant biological activities on specific diseases, including neurodegenerative diseases, certain types of cancer, gastrointestinal disease, and metabolic diseases, in which most of the interest has focused on ginsenoside Rd. The evidentiary basis showed that ginsenoside Rd ameliorates ischemic stroke, nerve injury, cancer, and other diseases involved in apoptosis, inflammation, oxidative stress, mitochondrial damage, and autophagy. In this review, we summarized available reports on the molecular biological mechanisms of ginsenoside Rd in neurological diseases, cancer, metabolic diseases, and other diseases. We also discussed the main biotransformation pathways of ginsenoside Rd obtained by fermentation.

## Graphical Abstract

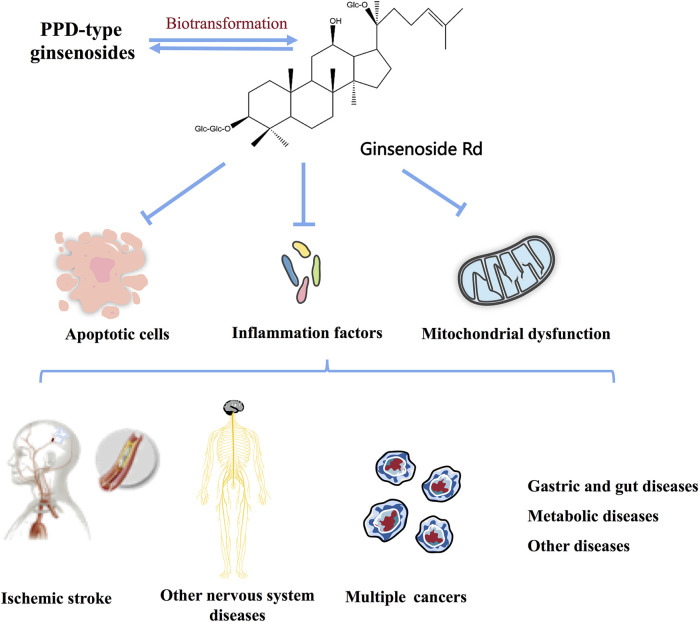

## Highlights

1) Approximately 120 studies on the use of ginsenoside Rd for the treatment of multiple diseases have been published.
2) This is the first review to report about the biotransformation, pharmacokinetics, and pharmacological effects of ginsenoside Rd.
3) The potential pharmacological mechanisms of ginsenoside Rd have been documented.
4) No specific reviews have been conducted by now.

## Introduction

*Panax ginseng* C.A. Mey. is a well-known herbal medicine widely used in China, Korea, Japan, and other East Asian countries. At present, the ginseng root and its extract are the most widely used herbal medicine. Modern pharmacological studies have proved that ginsenosides are the main active ingredient of ginseng and have a wide range of pharmacological effects, such as anti-inflammatory (Xu et al., 2021; Yi, 2021), anticancer (Zhang et al., 2021a), and anti-viral (Kang et al., 2021), regulate immunity (Kang et al., 2021), metabolism (Wang et al., 2021a), and improve cardiovascular system (Wang et al., 2021b; Sarhene et al., 2021) and nervous system (Brioschi Guevara et al., 2021) function, whereas most attention has been focused on the ginsenoside Rd.

Ginsenoside Rd, a natural compound extracted from the root of *Panax ginseng* C.A. Mey., is one of the protopanaxadiol (PPD)-type ginsenosides, while the proportion of ginsenoside Rd in ginseng is very low (Liu et al., 2020a). Interestingly, the promising effects of the pretreatment and treatment of ginsenoside Rd on neurological diseases, cancer, gastrointestinal disease, and metabolic diseases have been studied extensively in *in vivo* and *in vitro* models (Guo et al., 2021; Chen et al., 2022; Zhou et al., 2022).

Existing studies related to ginsenoside Rd have shown that various ginsenosides, such as Rb1, Rb2, and Rc, can be transformed into ginsenoside Rd after absorption and metabolism *in vivo* (Park et al., 2010; Shin and Oh, 2016). In addition, Rd can be prepared in a variety of ways based on the in-depth study of biotransformation and the development of modern fermentation technology (He et al., 2019). Based on the above results, we summarized the biotransformation process of other ginsenosides into Rd, thereby hoping to play a positive role in the large-scale industrial production of Rd. In this study, the biotransformation sources, pharmacokinetics, pharmacological effects, and molecular mechanisms of ginsenoside Rd on various systemic diseases in recent years were reviewed, and their therapeutic potential was discussed.

## Biotransformation of Ginsenoside Rd

**FIGURE 1:**
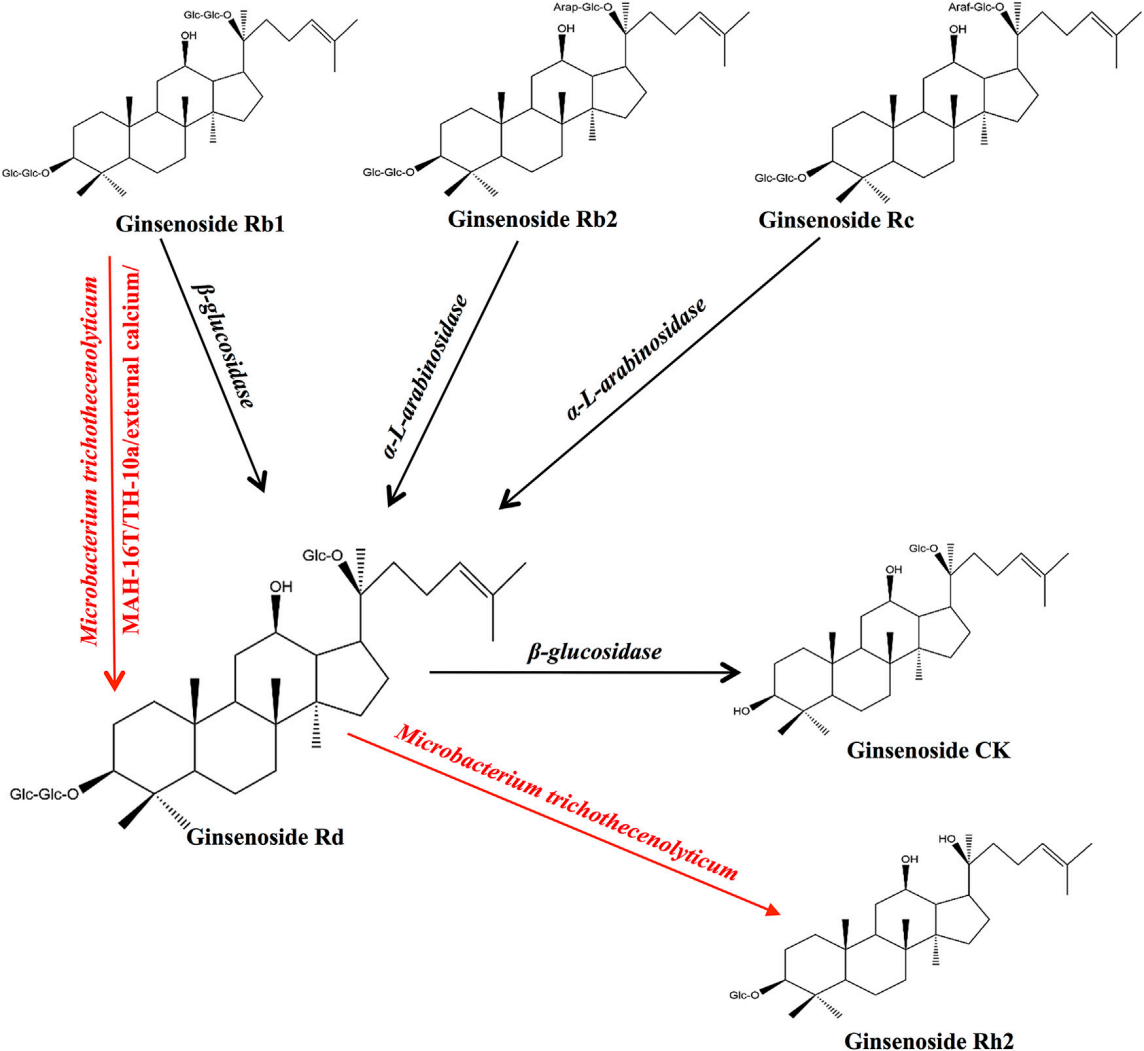
Biotransformation and pharmacokinetics of ginsenoside Rd *in vivo*.

Multiple studies have confirmed that ginsenosides can be transformed into ginsenoside Rd using enzymes and bacterial communities and can promote the transformation of ginsenoside Rd into other metabolites (He et al., 2019). We summarized the precursors, metabolites, and transformation conditions of ginsenoside Rd (Table 1) (Figure 1).

**TABLE 1:** Summary of the biotransformation of ginsenoside Rd.

References	Conversion	Source	Enzyme	Optimal conditions	Conversion ratio (%)
Akter and Huq, (2018)	Rb1 to Rd	*Paenibacillus*	MAH-16T	pH 5.0-7.0, 20-40°C	
Fang et al. (2020)	Rb1 to Rd		Pectinase	pH 6, 52.5°C	46.15
Renchinkhand et al. (2020)	Rb1 to Rd	*Dekkera anomala* YAE-1	β-glucosidase	pH 5.0, 40°C, 48 h	
Renchinkhand et al. (2017)	Rb1 to Rd	*Paenibacillus* sp. MBT213	β-glucosidase	pH7.0, 35°C, 14 days	
Hong et al. (2012)	Rb1 to Rd	*Flavobacterium johnsoniae*	β-glucosidase	pH 6.0, 37°C	
Quan et al. (2011)	Rb1 to XVII、Rd to F2 to CK	*Leuconostoc mesenteroides* DC102	Glucosidase	pH6.0–8.0, 30°C, 72 h	
Zhong et al. (2016)	Rb1 to Rd	*Lactobacillus brevis*	β-glucosidase	pH 7.0, 30°C	69
Feng et al. (2016)	Rb1 to Rd	*Aspergillus niger*	TH-10a	pH 5.0, 32°C, 48 h	86
Ye et al. (2010)	Rb1 to Rd	*Paecilomyces bainier* 229-7	β-glucosidase	pH5.0, 28°C, 72 h	89-94.9
Ye et al. (2012)	Rb1 to Rd	*Paecilomyces bainier* 229-7	External calcium regulated β-glucosidase	pH5.0, 28°C, 72 h	92.44
Son et al. (2008)	Rb1 to Rd	*Thermus caldophilus*	β-glucosidase	pH 5.0, 75°C, 18 h	80
Kim et al. (2013a)	Rb1 to Rd	*Microbacterium trichothecenolyticum*	*M.trichothecenolyticum* KCTC 19343	30°C, 24 h	
Zhao et al. (2009)	Rb1 to Rd	*Cladosporium fulvum*	β-glucosidase	pH 5.0, 37°C, 8 days	86
Lin et al. (2015)	Rb1 to Rd	*Aspergillus versicolor* LFJ1403	β-glucosidase	PH 5.0, 30°C, 96 h	85
Shin et al. (2013)	Rc to Rd	*Caldicellulosiruptor saccharolyticus* DSM 8903	α-L-arabinofuranosidase	pH 5.5, 80°C, 30 min	100
Xie et al. (2016a)	Rc to Rd	*Thermotoga thermarum* DSM5069	α-L-arabinofuranosidase	pH 5.0, 85°C, 60 min	99.4
Liu et al. (2013)	Rc to Rd	*Leuconostoc* sp. strain 22-3	α-L-arabinofuranosidase	pH 6.0, 30°C, 20 min	
Zhang et al. (2021b)	Rc to Rd	*Bacillus subtilis* Str. 168	α-L-arabinofuranosidase	pH 5, 40°C, 24 h	90
Kim et al. (2020)	Rb2 to Rd	*Blastococcus saxobsidens*	α-L-arabinopyranosidase	pH 7.0, 40°C, 1 h	
Jung et al. (2014)	F2 to Rd	Ginseng UDP-glycosyltransferases	UDP-glycosyltransferases 94Q2		

Ginsenoside Rd can be synthesized from ginsenoside Rb1 by the hydrolysis of glucose at C-20 (Akter and Huq, 2018). The β-glucosidase produced by pectinase (Fang et al., 2020), *Dekkera anomala* YAE-1 (Renchinkhand et al., 2020), *Paenibacillus* sp. MBT213 (Renchinkhand et al., 2017), *Flavobacterium johnsoniae* (Hong et al., 2012), *Leuconostoc mesenteroides* DC102 (Quan et al., 2011), and *Lactobacillus brevis* (Zhong et al., 2016) is able to hydrolyze ginsenoside Rb1 (Rb1) and convert it to ginsenoside Rd during the fermentation of the ginseng. In addition, *Aspergillus niger* strain TH-10 (Feng et al., 2016), *Paecilomyces bainier* 229-7 (Ye et al., 2010; Ye et al., 2012), *Thermus caldophilus* GK24 (Son et al., 2008), *Microbacterium trichothecenolyticum* (Kim et al., 2013a), *Cladosporium fulvum* (Zhao et al., 2009), and *Aspergillus versicolor* (Lin et al., 2015) have shown similar effects as those of hydrolases in Rb1.

The *α-L-arabinosidase* (AbpBs) from *Caldicellulosiruptor saccharolyticus* (Shin et al., 2013), *Thermotoga thermarum* DSM 5069 (Xie et al., 2016a), *Leuconostoc* sp. 22-3 (Liu et al., 2013), and *Bacillus subtilis* (Zhang et al., 2021b) converts ginsenoside Rc (Rc) into ginsenoside Rd by attacking the C-20 position of α-linked arabinoside, thereby releasing arabinose (Liu et al., 2013; Zhang et al., 2021b). AbpBs can promote the biotransformation of ginsenoside Rb2 (Rb2) to ginsenoside Rd by attacking C-20, thereby releasing arabinoside (Kim et al., 2020). In addition, enzymes PgUGT74AE2 and PgUGT94Q2, which participate in ginsenoside biosynthesis, transfer two glucose groups from UDP-glucose (UDP-Glc) to the C3 hydroxyl group of ginsenoside compound K (CK) to form ginsenoside Rd (Jung et al., 2014).

β-glucosidase cleaves the glycoside at the C-3 position of ginsenoside Rd and produces the ginsenoside compound CK (Renchinkhand et al., 2020). Ginsenoside M1 is formed by the hydrolysis of the C-3 glucose group in ginsenoside Rd by snailase (Renchinkhand et al., 2017).

## Pharmacokinetics

Intestinal flora can promote the metabolic transformation of ginseng extract and Rb1 into ginsenoside Rd in rats and can enter the blood for absorption in rats (Kim et al., 2014a). Ginsenoside Rd is distributed in various organs, with the highest content in the lungs, followed by the liver, kidney, heart, and intestine, and the lowest content in the brain (Sun et al., 2012). After taking urine 0–24 h after oral administration and intravenous administration, liquid chromatography-mass spectrometry (LC-MS) is used to confirm that oxidation and glycosylation (Yang et al., 2006a; Yang et al., 2007a) are the main metabolic pathways of ginsenoside Rd in rats. The absolute bioavailability of Rd in dogs is 0.26% (Wang et al., 2007). As in clinical trials, ginsenoside Rd shows linear pharmacokinetics, is well tolerated in the dose range of 10–75 mg after an intravenous administration, and is slowly cleared from plasma, and the elimination rate does not change after repeated administration (Zeng et al., 2010).

## Ginsenoside Rd Targets Multiple Diseases

### Ischemic Stroke

**FIGURE 2:**
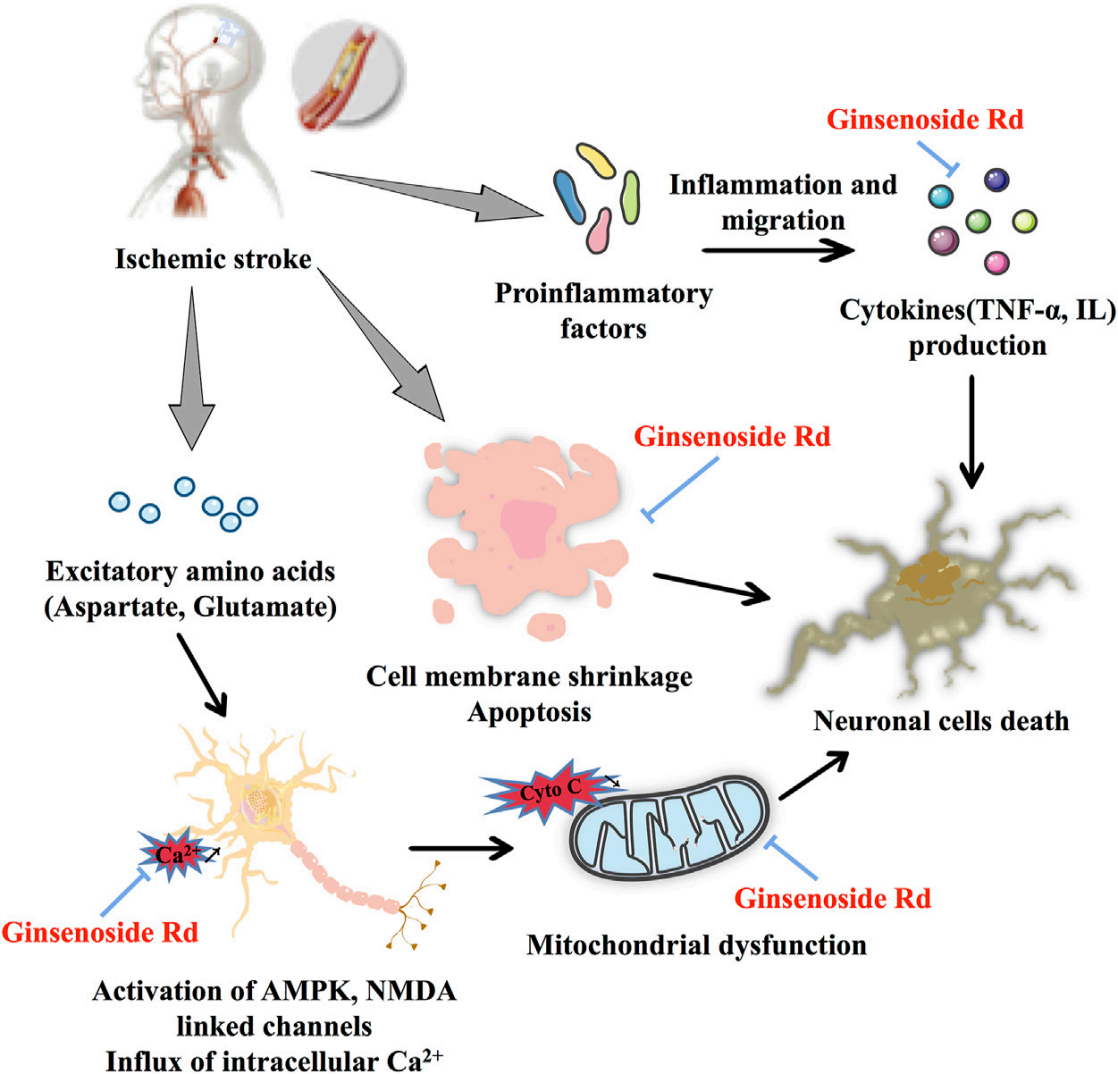
Protective effect of ginsenoside Rd on ischemic stroke.

In ischemic stroke, ginsenoside Rd plays a neuroprotective role by restoring mitochondrial function, reducing neuronal apoptosis, and eliminating neuroinflammation (Figure 2). As for the therapeutic window study, ginsenoside Rd shows an obvious neuroprotective effect in the middle cerebral artery occlusion (MCAO) model (Ye et al., 2011a). Importantly, the results of a clinical trial showed that ginsenoside Rd has a positive effect on the prognosis of acute ischemic stroke (Liu et al., 2012).

In Ca^2+^ influx and mitochondrial dysfunction, ginsenoside Rd, a potential Ca^2+^ channel blocker (Li et al., 2010), significantly reduces the burst of glutamate by increasing the expression of glutamate transporter-1 (GLT-1) and inhibits the channels of Ca^2+^ influx (Zhang et al., 2013a) to protect the rat hippocampal neurons (Zhang et al., 2012a). Similar to a calcineurin inhibitor, ginsenoside Rd exerts a neuroprotective effect by inhibiting the elevation of N-methyl-D-aspartate (NMDA) receptors and the hyperphosphorylation of the N-methyl-D-aspartate receptor 2B (NR2B) subunit in the MCAO model and oxygen–glucose deprivation (OGD) cultured neurons (Xie et al., 2016b; Zhang et al., 2020a). Ginsenoside Rd pretreatment exerts neuroprotective effects by inhibiting the Ca^2+^ overload and specificity attenuated the expression of transient receptor potential melastatin (TRPM) 7 and acid-sensing ion channel (ASIC) 1a while promoting ASIC2a expression following focal ischemia (Zhang et al., 2012b). Remarkably, the results of a clinical trial based on Ca^2+^ disorder and subsequent neurotoxicity induced by acuteischemic stroke, ginsenoside Rd can be considered a calcium channel antagonist and a neuroprotectant (Liu et al., 2009). As for mitochondrial dysfunction, ginsenoside Rd markedly protects the mitochondria, as indicated by regulating enzyme activity, reducing mitochondrial hydrogen peroxide production and depolarizing mitochondrial membrane potential (MMP), decreasing reactive oxygen species (ROS) production in isolated mitochondria from Sprague–Dawley (SD) rats (Ye et al., 2011b), and reducing the mitochondrial DNA (mtDNA) and nuclear DNA (nDNA) damage and cell apoptosis in MCAO-induced ischemic stroke model (Hu et al., 2013; Yang et al., 2016). These findings are also confirmed in primary cultured hippocampal neuron cells (Ye et al., 2009). In addition, in elderly stroke mice, ginsenoside Rd can play an equivalent neuroprotective role in elderly transient focal ischemic mice by regulating lipid peroxide accumulation, mitochondrial complex activity, and MMP (Ye et al., 2011c).

As far as apoptosis is concerned, ginsenoside Rd may reduce cerebral ischemia-induced tau phosphorylation by decreasing the activity of glycogen synthase kinase-3β (GSK-3β) and enhancing the activity of protein kinase B (PKB/AKT) (Zhang et al., 2014). In PC12 cells with OGD/reperfusion (OGD/R) and SD rats with ischemia/reperfusion (I/R) injury, ginsenoside Rd significantly limits the expression of vascular endothelial growth factor (VEGF), brain-derived neurotrophic factor (BDNF), and the phosphatidylinositol 3-kinase (PI3K)/AKT and ERK1/2 pathways (Liu et al., 2015a). As a neuroprotective agent ginsenoside Rd also prevents trimethyltin (TMT)-induced neurotoxicity and significantly reduces neuronal loss in TMT-induced hippocampal dysfunction and active astrocytes via regulation of B-cell lymphoma-2 (Bcl-2), Bcl-2-like protein 4, and caspase-3 (Hou et al., 2017). Taken together, ginsenoside Rd has neuroprotective effects via mitogen-activated protein kinase (MAPK)/ERK-, PI3K/AKT, PI3K/AKT/GSK-3β, and ERK1/2-dependent pathways.

For inflammation, ginsenoside Rd inhibits ischemic stroke-indeced neuronal death and inflammation by inhibiting cleaved poly adenosine diphosphate-ribose polymerase-1(PARP-1) activity, levels of poly (ADP-ribose), sequential apoptosis-inducing factor (AIF) translocation, and nuclear factor kappa-light-chain-enhancer of activated B cells (NF-κB) nuclear accumulation (Hu et al., 2013). Postischemic syntheses of two damaging enzymes, cyclooxygenase-2 (COX-2) and inducible nitric oxide synthase (iNOS), are also significantly inhibited by ginsenoside Rd treatment. Ginsenoside Rd reduces free radical generation during I/R and suppresses oxidative damage and inflammatory injury (Ye et al., 2011d). As a proteasome-related compound, ginsenoside Rd protects against MCAO-induced ischemic brain injury by inhibiting the proteasome activity and NF-κB/matrix metalloproteinase-9 (MMP-9) signal pathway (Zhang et al., 2020b). Ginsenoside Rd inhibits MCAO-induced microglial activation, decreases the expression levels of nuclear factor of kappa light polypeptide gene enhancer in B cell inhibitor, alpha (IκBα) phosphorylation and NF-κB nuclear translocation within a short time, and has fewer side effects than glucocorticoids (Zhang et al., 2016).

### Other Nervous System Diseases

Ginsenoside Rd has a significant neuroprotective effect on a variety of neurological diseases, which may be related to its promotion of stem cell proliferation (Shi et al., 2005) and differentiation into astrocytes (Lin et al., 2012). Ginsenoside Rd may promote neurite outgrowth by upregulating growth-associated protein of 43 kDa (GAP-43) expression via ERK- and ARK-dependent signaling pathways in NGF-induced PC12 cells (Wu et al., 2016).

In H_2_O_2_-induced PC12 cells, ginsenoside Rd, as a neuroprotective agent, has neuroprotective effects on neurodegenerative diseases (Ye et al., 2008). In the converting monocyte phenotype and macrophages of the Guillain–Barre syndrome (GBS) mouse model, ginsenoside Rd attenuates experimental autoimmune neuritis (Ren et al., 2021). Ginsenoside Rd can regulate MMP by decreasing intracellular ROS and enhancing the activity of antioxidant enzymes and mitochondrial complex, thereby increasing intracellular ATP levels and ultimately reducing 1-methyl-4-phenylpyridinium (MPP^+^)-induced cell death in Parkinson’s disease (PD) (Liu et al., 2015b). Meanwhile, in the Aβ_25–35_-induced neuronal damage model, apoptosis and oxidative stress are ameliorated by ginsenoside Rd by regulating antioxidant capacity and the production of apoptotic proteins (Liu et al., 2015c). Learning and memory abilities can be improved in ginsenoside Rd-pretreated APP transgenic mice by significantly suppressing the NF-κB pathway to reduce the generation of proinflammatory factors (Liu et al., 2015d). Ginsenoside Rd-mediated neuroprotective effects against Alzheimer’s disease (AD) progression play a significant role in Neuro2a cells (Kim et al., 2014b). Ginsenoside Rd pretreatment can inhibit tau protein phosphorylation by maintaining a balance of GSK-3β, cyclin-dependent kinase 5 (CDK5/P25), and protein phosphatase 2A (PP-2A) (Li et al., 2013) to inhibit tau phosphorylation of tau protein at Ser199/202, Ser396, or Ser404 in okadaic acid-induced rats, APP transgenic mice, and cortical neurons to increase PP-2A activity for protection against AD (Li et al., 2011a; Li et al., 2021), respectively. Moreover, ginsenoside Rd increases the soluble amyloid-β precursor protein α (sAPPα) level and reduces extracellular Aβ to enhance the cognitive and memory functions of ovariectomy rats (Yan et al., 2017).

In experimental autoimmune encephalomyelitis, ginsenoside Rd exerts a neuroprotective role by regulating the immune response and inflammatory reaction via a signal pathway of IFN-g/IL-4, BDNF/NGF (Zhu et al., 2014), and Foxp3/RORγt/JAK2/STAT3 (Jin et al., 2020a). In spinal cord injury (SCI) models, ginsenoside Rd shows anti-inflammatory effects consistent with dexamethasone that could significantly decrease the biomarkers of apoptosis, inflammation, oxidative damage factor, and repaired damaged mitochondria; particularly, there is no obvious difference in terms of dexamethasone in anti-inflammatory (Zhou et al., 2014; Cong and Chen, 2016), and these effects depended on the ASK1/JNK pathway (Wang et al., 2014). In the pathology of noise-induced hearing loss (NIHL), ginsenoside Rd could alleviate the apoptosis and oxidative stress damage on neuron cells by activating the SIRT1/PGC-1α signaling pathway (Chen et al., 2020). In addition, ginsenoside Rd treatment effectively eliminates the oxidative injury and the production of proinflammatory factors and peroxides in the chronic restraint stress (CRS) paradigm (Wang et al., 2020). Ginsenoside Rd pretreatment may be neuroprotective in old rats following acute Pb exposure through limited microglial activation and maintained neural stem cell proliferation (Wang et al., 2013a).

To summarize, ginsenoside Rd can play a significant role in neuron damage by inhibiting the production of excitatory amino acids, reducing the intracellular Ca^2+^ influx mediated by the NMDA pathway, changing the neurotoxicity of Ca^2+^ to mitochondrial function damage, and regulating apoptosis-inducing and neuroinflammatory factors (Table 2).

**TABLE 2:** Summary of the neuroprotective effects and mechanism of ginsenoside Rd in animal and cell models.

References	Diseases	Inducer	Experimental model	Effects	Mechanism
Zhang et al. (2013a)	Ischemic stroke	MCAO	Male SD rats	GLT-1, PKB/Akt, p-ERK1/2↑	Glutamate metabolism
				Glutamate↓	
Zhang et al. (2012a)	Ischemic stroke	Glutamate, NMDA	Primary hippocampal cell cultures from SD rat embryos	TUNEL-positive cells, caspase-3, Ca^2+^↓	Ca^2+^, apoptosis
Xie et al. (2016b)	Stroke	OGD/Transient MCAO	Adult male primary cortical neuron cells/SD rats	Infarct volume, NR2B subunit, p-Ser-1303, p-Tyr-1472, p-Tyr-1480↓	Hyperphosphorylation of neurons
Zhang et al. (2020a)	Ischemic stroke	OGD/MCAO, CsA	Primary cortical neurons cells, HEK293 cells/Adult male SD rats	Ca^2+^, NMDA receptor currents, caspase3↓	Apoptosis
Zhang et al. (2012b)	Ischemic stroke	MCAO	Male SD rats	ASIC2a↑	Ca^2+^ overload
				TRPM7, ASIC1a↓	
Ye et al. (2011b)	Transient ischemic stroke	MCAO	Male SD rats, isolated mitochondria	ETC, aconitase, MMP, Pyruvate↑	Mitochondrial dysfunction, apoptosis
				ROS, Lactate, caspase-3, Cyto C, AIF↓	
Yang et al. (2016)	Ischemic stroke	MCAO	Male SD rats	NEIL1, NEIL3↑	mtDNA and nDNA damages, apoptosis
				Cleaved caspase-3↓	
Hu et al. (2013)	Cerebral ischemia	MCAO	Adult male SD rats	PARP-1, NF-κB, AIF↓	Apoptosis, inflammation
Ye et al. (2009)	Cerebral ischemic injury	OGD	Primary hippocampal neurons cells	GSH, GPX,SOD,CAT,MMP↑	Oxidative stress, apoptosis
				ROS, MDA,LDH, GSSG↓	
Ye et al. (2011c)	Transient focal ischemia in the aged brain	MCAO	Male C57BL/6 mice	Mitochondrial complex, MMP, CAT, SOD, GPX, GST↑	Mitochondrial dysfunction oxidative stress
				MDA, protein carbonyl concentration, ROS, mitochondrial aconitase↓	
Zhang et al. (2014)	Ischemic stroke	OGD/MCAO	Primary culture of neurons/Male SD rats	p-AKT, GSK-3β↑	p-tau
				p-tau, S199/202, PHF-1↓	
Liu et al. (2015a)	Stroke	OGD/R/Transient MCAO followed by reperfusion	PC12 cells/Male SD rats	p-AKT, p-ERK, VEGF, BDNF↑	Apoptosis
Hou et al. (2017)	TMT intoxication	Trimethyltin	Primary hippocampal neuron/Male ICR mice	Bcl-2↑	Apoptosis
				Bax, caspase-3↓	
Ye et al. (2011d)	Transient ischemic stroke	MCAO	Male SD rats	CAT, SOD 1 and 2, GR, GSH/GSSG↑	Oxidative stress, inflammation
				2,3- and 2,5-DHBA, 8-OHdG positive cells, 4-HNE, MDA, AGEs↓	
Zhang et al. (2020b)	Transient forebrain ischemia	MCAO	Male SD rats	IκB-α↑	Inflammation
				20S proteasome, NF-κB, p65, matrix MMP-9↓	
Zhang et al. (2016)	Ischemic stroke	OGD or LPS/MCAO	BV2 cells/Adult male SD rats	IL-1β, IL-6, TNF-α, IFN-γ, p-IκBα↓	Inflammation
Wu et al. (2016)	Ischemic stroke	NGF	PC12 cells	p-ERK1/2, p-AKT	NGF
				GAP-43↑	
Ye et al. (2008)	Oxidative damage	H_2_O_2_	PC12 cells	SOD, GPX, MMP↑	Oxidative stress, mitochondrial dysfunction
				LDH, ROS, MDA,↓	
Ren et al. (2021)	GBS	Peripheral nerve antigen P0_180–199_ peptide, Pertussis toxin (PTX)	Male C57 BL/6 mice	Non-classical Ly6C^lo^ monocytes	Immunization, inflammation
				Nr4a1↑	
				IL-12, IL-1β, TNF- α, IL-6, CD45+Ly6G ^+^↓	
Liu et al. (2015b)	Parkinson disease	MPP ^+^	SH-SY5Y cells/C57BL/6J mice	SOD, GPX, MMP, complex I, ATP, Bcl-2, p-Akt↑	Oxidative stress, mitochondrial dysfunction
				LDH, ROS, MDA, Bax↓	
Liu et al. (2015c)	Alzheimer’s disease	Aβ_25-35_	Primary cultured hippocampal neurons cells	SOD, GSH-Px, Bcl-2 mRNA↑	Oxidative stress, Neuronal apoptosis
				ROS, Bax mRNA, Caspase-3, Cyt C mRNA↓	
Liu et al. (2015d)	Alzheimer’s disease		APP transgenic mice	IL-1β, IL-6, TNF-α, S100β mRNA, NF-κB p65↓	Inflammation
				IL-10↑	
Kim et al. (2014b)	Neurodegenerative diseases		Neuro2a cells	ChAT, VAChT, ACh, MAP-2, p75, p21, TrkA↑	Cholinergic markers
Li et al. (2013)	Alzheimer’s disease		APP transgenic mice	Ser9, PP-2A↑	p-tau
				GSK-3β, Tyr216↓	
Li et al. (2011a)	Alzheimer’s disease	Okadaic Acid	Adult male SD rats/Cortical neurons cells	PP-2A↑	Tau
				Tau↓	
Li et al. (2021)	Alzheimer’s disease		APP transgenic mice	P35↑	p-tau
				Tau, P25↓	
Yan et al. (2017)	Alzheimer’s disease	Ovariectomy/Inhibitor	Adult female rats/HT22 hippocampal neuronal cells	BACE1, Aβ↓ sAPPα, ADAM↑	Activating estrogen-like activity
Zhu et al. (2014)	Multiple sclerosis	Experimental autoimmune encephalomyelitis	6-8 weeks female C57 BL/6 mice	IL-4, BDNF, NGF↑	Blood–brain barrier, inflammation
				IFN-γ↓	
Jin et al. (2020a)	Multiple sclerosis	Experimental autoimmune encephalomyelitis	Splenocyte/6-8 weeks C57BL/6 mice	TGF-β, IL-10, Treg, Foxp3↑	Inflammation, autoimmunity
				IL-6, IL-17, RORγt, Jak1, Jak2,STAT↓	
Cong and Chen, (2016)	Spinal cord injury	T8 laminectomy and a spinal contusion injury	Adult female SD rats	MDA, TNF-α, IL-1β, IL-6, Bax, GSK, SOD, Bcl-2↑	Oxidative stress, inflammation, apoptosis
				cleaved-caspase 3, p-ERK, p-JNK, p-p38↓	
Zhou et al. (2014)	Paraplegia	Ca^2+^	Isolated spinal cord mitochondria/Male C57BL/6J mice	p-AKT, p-ERK↑	Mitochondrial dysfunction
				Cyto C↓	
Wang et al. (2014)	Delayed paralysis	Occlusion of the abdominal aorta for 1 h	Female SD rats	Caspase 3, ASK1, JNK↓	Apoptosis
Wang et al. (2020)	Cognitive impairment	Respiration in a transparent plexiglas restrainer with many air holes to for 10 h	Male C57BL/6J mice	SOD, CAT, GSH, GPX, p-PI3K, p-CREB, BDNF, TrkB↑	Oxidative stress, inflammation, neurotrophic factors
				TNF-α, IL-6, p-AKT↓	
Wang et al. (2013a)		Lead (Pb) exposure	Retired breeder SD rats	IL-1β, IL-6, TNF-α↓	Inflammation

Abbreviations: CsA, cyclosporin A; ETC, mitochondrial electron transport chain; CAT, catalase; SOD, superoxide dismutase; GPX, glutathione peroxidase; GR, glutathione reductase; GSH, glutathione; GSSG, glutathione disulfide; 8-OHdG, 8-hydroxy-deoxyguanosine; 4-HNE, 4-hydroxynonenal; MDA, malondialdehyde; AGEs, advanced glycosylation end products; NGF, nerve growth factor; PTX, pertussis toxin; Nr4a1, nuclear receptor subfamily 4 group A member 1; ChAT, choline acetyltransferase; VAChT, vesicular acetylcholine transporter; ACh, acetylcholine.

### Cancer

**FIGURE 3:**
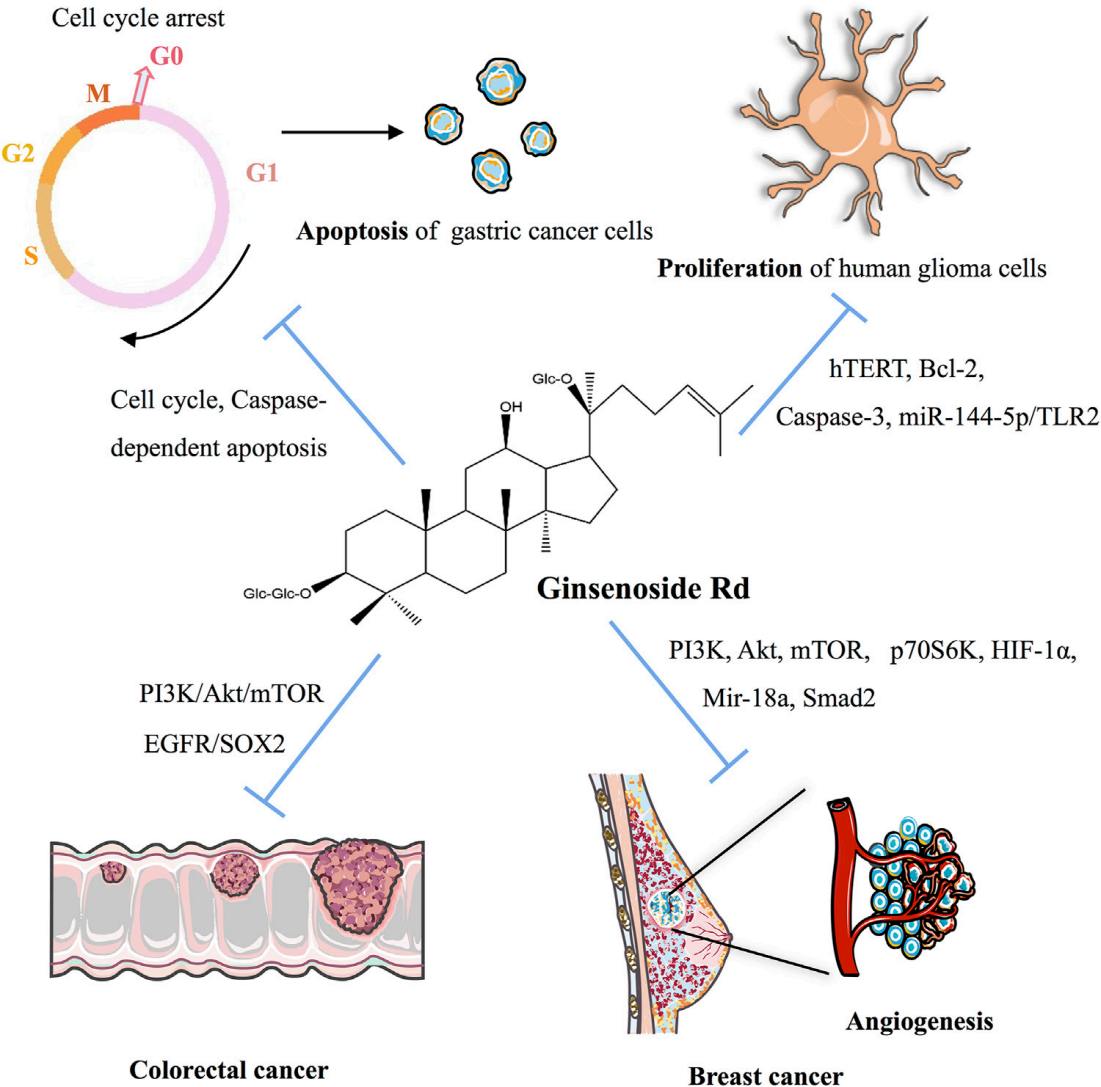
Molecular mechanism of ginsenoside Rd on anticancer activity in multiple cancers.

As indicated in Table 3 and Figure 3, ginsenoside Rd can inhibit the proliferation of various cancer cells by participating in the apoptotic pathway. As a potential therapeutic and specific 26S proteasome inhibitor, ginsenoside Rd plays an important role in anticancer therapy by targeting 26S proteasome (Chang et al., 2008).

**TABLE 3:** Summary of the effects and mechanisms of ginsenoside Rd on cell and animal models of multiple cancers.

References	Diseases	Experimental model	Effects	Mechanism
Tian et al. (2020)	Gastric cancer	MKN-45, SGC-7901 cells	Caspase-3, caspase-9↑	Apoptosis
			Cyclin D1↓	
Kim et al. (2013b)	Gastric cancer	AGS cells	Caspase-3, caspase8, PARP↑	Apoptosis
Chian et al. (2019)	NSCLC	A549 NSCLC cells	NRF2↓	Proliferation
Gu et al. (2019)	Glioblastoma	U251 cells	Caspase‐3↑	Apoptosis
			Bcl‐2, hTERT↓	
Liu et al. (2020b)	Glioblastoma	U251 cells, H4 (HTB148) cells, U87 MG cells	miR-144-5p, TLR2↑	Proliferation
			Toll-like receptor 2↓	
Phi et al. (2019)	Colorectal cancer	Human CRC cell, HT29 cells/SW620, NSG mice	Smad2↓	Apoptosis
Zhang et al. (2017)	Breast cancer	HUVECs, MDA-MB-231 cells/Athymic nude mice	Bax, caspase-3, HIF1-α↑	Apoptosis
			Bcl-2↓	
Kim, (2013)	Breast cancer	AGS cells, MCF-7 cells	Caspase-3↑	Apoptosis
Wang et al. (2016)	Breast cancer	4T1 cells, MDA-MB-231cells/Female BALB/c mice	Smad2↑	Attenuates metastasis
			miR-18a↓	
Pokharel et al. (2010)	Breast cancer	MCF-7/ADR cells	MDR1↓	Resistance
Yang et al. (2006b)	Cervical cancer	HeLa cells	Bax↑	Apoptosis
			Bcl-2↓	
Yang et al. (2021a)	Hepatocellular carcinoma	HepG2 cells/Male BALB/c nude mice		Proliferation, apoptosis
Yoon et al. (2012)	Hepatocellular carcinoma	HepG2 cells	MMP-1, MMP-2, MMP-7↓	Blocking MAPK signaling and inducing the formation of focal adhesions

Ginsenoside Rd can appreciably inhibit the proliferation of gastric cancer cells and can stimulate apoptosis by downregulating cyclin D1, thereby inducing cell cycle arrest in the G0/G1 phase and enhancing the expression of caspase-3 and caspase-9 and the ratio of Bax/Bcl-2 (Tian et al., 2020). After heat processing, the anticancer activity of deglycosylated Rd could be improved via the apoptotic pathway for AGS cells (Kim et al., 2013b).

In non-small-cell lung cancer (NSCLC), ginsenoside Rd, as a therapeutic drug, inhibits the nuclear factor erythroid 2-associated factor 2 (NRF2) pathway, and the synergistic effect of ginsenoside Rd in A549 and cisplatin (DDP)-resistant A549 cell lines (A549/DDP) can be weakened by knocking out NRF2 (Chian et al., 2019). As for glioblastoma, ginsenoside Rd decreases the proliferation of human glioma U251 cells and promotes apoptosis by downregulating the expression of hTERT and Bcl-2, upregulating the expression of the caspase-3 level, and inhibiting the telomerase activity of U251 cells (Gu et al., 2019). Ginsenoside Rd inhibits the proliferation and migration of glioblastoma cells by decreasing the expression of tumor-suppressor Mir-144-5p and promoting the expression of the target of Mir-144-5p toll-like receptor 2 (Liu et al., 2020b). In colorectal cancer cells, ginsenoside Rd, a therapeutic agent, targets epidermal growth factor receptor (EGFR)/SOX2 signaling (Phi et al., 2019).

Ginsenoside Rd also plays a crucial role in breast cancer. In MDA-MB-231 cell xenografted mice, ginsenoside Rd treatment inhibits the activation of PI3K, AKT, mammalian target of rapamycin (mTOR), and p70S6K in cells and decreases the expression of hypoxia-inducible factor 1-α (HIF1-α) (Zhang et al., 2017). In MCF-7 cells, ginsenoside Rd inhibits the proliferation of MCF-7 cells by enhancing caspase-3 activity, mitochondrial depolarization, and sub-G1 populations (Kim, 2013). In 4T1 cells, the expression of Mir-18a and Smad2 decreases with ginsenoside Rd treatment (Wang et al., 2016). Furthermore, ginsenoside Rd promotes the ubiquitination of MDR1 and inhibits doxorubicin resistance in MCF-7/ADR cells (Pokharel et al., 2010). In cervical cancer, ginsenoside Rd treatment in HeLa cells upregulates Bax expression, downregulates Bcl-2 expression, decreases the mitochondrial transmembrane potential, activates the caspase-3 pathway, significantly inhibits proliferation, and induces apoptosis (Yang et al., 2006b).

Finally, in HepG2 cells and the HepG2 cell-injected nude mice-induced hepatocellular carcinoma model, the combination of CA4P and ginsenoside Rd has synergistic antitumor effects via the PI3K/AKT/mTOR signaling pathway-related inhibition of HIF-1α (Yang et al., 2021a). HepG2 cells treated with ginsenoside Rd noticeably promoted matrix metalloproteinases’ (MMPs) activation, and MAPK signaling pathways were involved in cancer cell migration, thereby suggesting that ginsenoside Rd inhibits the activity of HepG2 cells in a dose-dependent and time-dependent manner (Yoon et al., 2012).

### Gastric and Gut

In a sodium dextran sulfate (DSS)-induced colitis model, ginsenoside Rd reduces DSS-induced colonic pathology via the adenosine 5′-monophosphate-activated protein kinase/Unc-51 like autophagy activating kinase 1 (AMPK/ULK1)-induced autophagy signaling pathway and the inhibition of the production of proinflammatory cytokines (IL-1β, TNF-α, and IL-6) in serum and colon tissues (Liu et al., 2018). In irradiation-induced intestinal epithelial cells, ginsenoside Rd reduces apoptosis by activating a pathway of PI3K/AKT, inactivates MEK, and inhibits a mitochondria/caspase pathway (Tamura et al., 2008). Meanwhile, in 2,4,6-trinitrobenzenesulfonic acid (TNBS)-induced ulcerative colitis model, ginsenoside Rd showed obvious anti-inflammatory activity by inhibiting neutrophil infiltration, regulating apoptosis signal and oxidative stress (Yang et al., 2012a), reduced the accumulation of leukocytes, and downregulated multiple proinflammatory cytokines (Yang et al., 2012b).

### Metabolic Diseases

Laboratory data of ginsenoside Rd suggest that it has effects on multiple metabolic diseases. The browning of white adipose tissue induced by cold stress and cAMP levels are increased by ginsenoside Rd. In particular, Rd alleviates obesity and insulin resistance by upregulating thermogenesis through the cAMP/protein kinase A (PKA) signaling pathway (Yao et al., 2020). In fast-food diet-induced non-alcoholic fatty liver disease (NAFLD), fermented ginsenoside Rd with *Cordyceps militaris* regulates lipid metabolism and the inflammatory response via mTORC1 signaling (Choi et al., 2019). Ginsenoside Rd inhibits the progress of the death of islet transplantation by decreasing the apoptosis of the islet cells (Kaviani et al., 2019). In the atherosclerosis process, ginsenoside Rd decreases oxidized low-density lipoprotein (Ox-LDL) and cholesterol by inhibiting Ca^2+^ influx (Li et al., 2011b). In diabetic db/db mice and mesangial cells, pectin-lyase-modified ginsenoside Rd relieves diabetic nephropathy via alleviated ROS production (Jung et al., 2021).

### Other Diseases

Ginsenoside Rd has positive effects on skin injury, osteoporosis, kidney injury, vessel injury, heart injury, lung injury, aging, and inflammation. In animal wound models, ginsenoside Rd significantly increases wound healing by promoting the proliferation and migration level of keratinocyte progenitor cells (KPCs) and human dermal fibroblasts (HDFs) (Kim et al., 2013c). Ginsenoside Rd also has a positive effect on rejection caused by a transplant skin allograft (Wang et al., 2012a). Beyond that, ginsenoside Rd, as an antiosteoporotic agent, promotes differentiation and mineralization in osteoblastic MC3T3-E1 cells (Kim et al., 2012). In animal models of renal I/R injury and cultured proximal tubule cells, ginsenoside Rd has a protective effect by inhibiting inflammation and regulating biochemical indexes of renal function (Yokozawa et al., 1998; Ren et al., 2016). In addition, ginsenoside Rd downregulates NF-κB and the expression of iNOS and COX-2 in lipopolysaccharide (LPS)-induced Institute of Cancer Research (ICR) mice, and RAW264.7 cells were suppressed (Kim et al., 2013d). In the nicotine-induced vascular endothelial injury model, ginsenoside Rd plays an important role in the prevention of cardiovascular diseases via participation in NO signaling and regulates platelet and vascular function (Zhang et al., 2020c). Ginsenoside Rd upregulates Cyto C release and caspase-9/caspase-3 activation and decreases the MMP and the ratio of Bcl-2/Bax via the mitochondria-dependent pathway in H_2_O_2_-induced apoptosis in basilar artery smooth muscle cells (BASMCs) (Li et al., 2012). Furthermore, ginsenoside Rd could relieve the cisplatin-induced kidney injury (Yokozawa and Liu, 2000; Yokozawa and Dong, 2001) and kidney proximal tubules cephaloridine injury under cephaloridine treatment (Yokozawa and Dong, 2001). In an adrenocorticotrophic hormone (ACTH)–induced corticosterone secretion cell model, ginsenoside Rd inhibits ACTH-induced corticosterone production by inhibiting the MC2R-cAMP/PKA/cyclic AMP response element binding (CREB) pathway in adrenocortical cells (Jin et al., 2020b). In myocardial I/R-induced rats and simulated I/R-induced primary neonatal rat cardiomyocyte models, ginsenoside Rd promotes cardioprotection via the activation of AKT/GSK-3b signaling (Wang et al., 2013b). In addition, ginsenoside Rd can protect against LPS-induced acute lung injury by inhibiting the PI3K/AKT signaling pathway (Yang et al., 2021b). Other studies have indicated that ginsenoside Rd can significantly enhance the survival time of *Caenorhabditis elegans* via lipid metabolism and the activation of the stress response signaling pathway (Yu et al., 2021) and can alleviate the oxidative damage caused by aging in senescence-accelerated mice (Yokozawa et al., 2004). Finally, the anti-inflammatory activity of ginsenoside Rd is well documented, is considered to be associated with its antioxidant effects (Kim et al., 2007; Zhang et al., 2013b), and selectively produces prostaglandin E2 (PGE2) by activating the CCAAT/enhancer binding protein (C/EBP) and CREB to express COX-2 (Jeong et al., 2007). Ginsenoside Rd exerts anti-inflammation effects in carrageenan-induced inflammation rats via the inhibition of the NF-κB signaling pathway (Wang et al., 2012b) and in ovalbumin-induced allergic rhinitis mice by regulating multiple inflammatory factors (Kim et al., 2019) and elicits a Th1 and Th2 immune responses (Yang et al., 2007b). Ginsenoside Rd enhances the Th1 response to surface mannan extract in mice, which protects mice from disseminated *candida* infection by stimulating higher titers of Th1 antibodies and a Th1-dominated immune response (Han and Rhew, 2013).

## Conclusion and Perspective

As a widely used herbal medicine, ginseng appears in the form of dietary supplements nowadays. Available evidence suggests that the antiapoptotic, antioxidant, and anti-inflammatory activities, which suppress the calcium influx of ginsenoside Rd, may have an important role in the neuroprotective and anticancer effects. Ginsenoside Rd play a crucial role in neuroprotective, anticancer effects, metabolism, and other diseases by regulating PI3K/AKT, inhibiting Cyto C released and caspase activation, and regulating the release of inflammatory factors, which play a crucial role in neuroprotective, anticancer effects, metabolism, and other diseases.

In addition, ginsenoside Rd has potential therapeutic effects on regulating metabolism and in multiorgan protection. However, attributable to the shortage of clinical studies on ginsenoside Rd, it is difficult to make a clear decision. In addition to exploring its various activities, it is suggested to verify existing activities in a deeper mechanism, design clinical trials to prove its safety and effectiveness, and obtain a more extensive clinical application.

## Glossary

PPD: protopanaxadiol
LC-MS: liquid chromatography-mass spectrometry
MCAO: middle cerebral artery occlusion
GLT-1: glutamate transporter-1
NMDA: N-methyl-D-aspartate
NR2B: N-methyl-D-aspartate receptor 2B
OGD: oxygen–glucose deprivation
TRPM: transient receptor potential melastatin
ASIC: acid-sensing ion channel
MMP: mitochondrial membrane potential
ROS: reactive oxygen species
SD: Sprague–Dawley
mtDNA: mitochondrial DNA
nDNA: nuclear DNA
PKB: protein kinase B: protein kinase B
GSK-3β: glycogen synthase kinase-3β
PKB: protein kinase B: protein kinase B
OGD/R: oxygen–glucose deprivation/reperfusion
I/R: ischemia/reperfusion
VEGF: vascular endothelial growth factor
BDNF: brain-derived neurotrophic factor
PI3K: phosphatidylinositol 3-kinase
TMT: trimethyltin
Bcl-2: B-cell lymphoma-2
MAPK: mitogen-activated protein kinase
PARP-1: poly adenosine diphosphate-ribose polymerase-1
AIF: apoptosis-inducing factor
NF-κB: nuclear factor kappa-light-chain-enhancer of activated B cells
COX-2: cyclooxygenase-2
iNOS: inducible nitric oxide synthase
MMP-9: matrix metalloproteinase-9
IκBα: nuclear factor of kappa light polypeptide gene enhancer in B cells inhibitor, alpha
GAP-43: growth-associated protein of 43 kDa
GBS: Guillain–Barre syndrome
MPP+: 1-methyl-4-phenylpyridinium
PD: Parkinson’s disease
AD: Alzheimer’s disease
CDK5/P25: cyclin dependent Kinase 5
PP-2A: protein phosphatase 2A
sAPPα: soluble amyloid-β precursor protein α
SCI: spinal cord injury
NIHL: noise-induced hearing loss
CRS: chronic restraint stress
NSCLC: non-small-cell lung cancer
NRF2: nuclear factor erythroid 2-associated factor 2
DDP: cisplatin
EGFR: epidermal growth factor receptor
mTOR: mammalian target of rapamycin
HIF1-α: hypoxia-inducible factor 1-α
MMPs: matrix metalloproteinases
DSS: sodium dextran sulfate
AMPK: adenosine 5′-monophosphate-activated protein kinase
ULK1: Unc-51 like autophagy activating kinase 1
TNBS: trinitrobenzenesulfonic acid
PKA: protein kinase A
NAFLD: non-alcoholic fatty liver disease
Ox-LDL: oxidation low lipoprotein
KPCs: keratinocyte progenitor cells
HDFs: human dermal fibroblasts
LPS: lipopolysaccharides
ICR: Institute of Cancer Research
BASMCs: basilar artery smooth muscle cells
ACTH: adrenocorticotrophic hormone
PGE2: prostaglandin E2
C/EBP: CCAAT/enhancer binding protein
CREB: cyclic AMP response element binding protein

## References

[B1] Akter S. Huq M. A. (2018). Biological Synthesis of Ginsenoside Rd Using Paenibacillus Horti Sp. Nov. Isolated from Vegetable Garden. Curr. Microbiol. 75, 1566–1573. 10.1007/s00284-018-1561-6 30167766

[B2] Brioschi Guevara A. Bieler M. Altomare D. Berthier M. Csajka C. Dautricourt S. (2021). Protocols for Cognitive Enhancement. A User Manual for Brain Health Services-Part 5 of 6. Alz Res. Ther. 13, 172. 10.1186/s13195-021-00844-1 PMC8507160 34635149

[B3] Chang T. L. Ding H. Y. Kao Y. W. (2008). Role of Ginsenoside Rd in Inhibiting 26S Proteasome Activity. J. Agric. Food Chem. 56, 12011–12015. 10.1021/jf801427e 19053398

[B4] Chen X. M. Ji S. F. Liu Y. H. Xue X. M. Xu J. Gu Z. H. (2020). Ginsenoside Rd Ameliorates Auditory Cortex Injury Associated with Military Aviation Noise-Induced Hearing Loss by Activating SIRT1/PGC-1α Signaling Pathway. Front. Physiol. 11, 788. 10.3389/fphys.2020.00788 32792971 PMC7385399

[B5] Chen Y. Y. Liu Q. P. An P. Jia M. Luan X. Tang J. Y. (2022). Ginsenoside Rd: A Promising Natural Neuroprotective Agent. Phytomedicine 95, 153883. 10.1016/j.phymed.2021.153883 34952508

[B6] Chian S. Zhao Y. Xu M. Yu X. Ke X. Gao R. (2019). Ginsenoside Rd Reverses Cisplatin Resistance in Non-small-cell Lung Cancer A549 Cells by Downregulating the Nuclear Factor Erythroid 2-related Factor 2 Pathway. Anticancer Drugs 30, 838–845. 10.1097/CAD.0000000000000781 31415285

[B7] Choi S. Y. Park J. S. Shon C. H. Lee C. Y. Ryu J. M. Son D. J. (2019). Fermented Korean Red Ginseng Extract Enriched in Rd and Rg3 Protects against Non-alcoholic Fatty Liver Disease through Regulation of mTORC1. Nutrients 11. 10.3390/nu11122963 PMC6949916 31817227

[B8] Cong L. Chen W. (2016). Neuroprotective Effect of Ginsenoside Rd in Spinal Cord Injury Rats. Basic Clin. Pharmacol. Toxicol. 119, 193–201. 10.1111/bcpt.12562 26833867

[B9] Fang H. Wei Y. Li Y. Zhou G. (2020). One-Pot Process for the Production of Ginsenoside Rd by Coupling Enzyme-Assisted Extraction with Selective Enzymolysis. Biol. Pharm. Bull. 43, 1443–1447. 10.1248/bpb.b19-01127 32999154

[B10] Feng L. Xu C. Li Z. Li J. Dai Y. Han H. (2016). Microbial Conversion of Ginsenoside Rd from Rb1 by the Fungus Mutant Aspergillus niger Strain TH-10a. Prep. Biochem. Biotechnol. 46, 336–341. 10.1080/10826068.2015.1031391 25831478

[B11] Gu B. Wang J. Song Y. Wang Q. Wu Q. (2019). The Inhibitory Effects of Ginsenoside Rd on the Human Glioma U251 Cells and its Underlying Mechanisms. J. Cell Biochem. 120, 4444–4450. 10.1002/jcb.27732 30260020

[B12] Guo Y. X. Zhang Y. Gao Y. H. Deng S. Y. Wang L. M. Li C. Q. (2021). Role of Plant-Derived Natural Compounds in Experimental Autoimmune Encephalomyelitis: A Review of the Treatment Potential and Development Strategy. Front. Pharmacol. 12, 639651. 10.3389/fphar.2021.639651 34262447 PMC8273381

[B13] Han Y. Rhew K. Y. (2013). Ginsenoside Rd Induces Protective Anti-Candida Albicans Antibody through Immunological Adjuvant Activity. Int. Immunopharmacol. 17, 651–657. 10.1016/j.intimp.2013.08.003 24007781

[B14] He Y. Hu Z. Li A. Zhu Z. Yang N. Ying Z. (2019). Recent Advances in Biotransformation of Saponins. Molecules 24. 10.3390/molecules24132365 PMC6650892 31248032

[B15] Hong H. Cui C. H. Kim J. K. Jin F. X. Kim S. C. Im W. T. (2012). Enzymatic Biotransformation of Ginsenoside Rb1 and Gypenoside XVII into Ginsenosides Rd and F2 by Recombinant β-glucosidase from Flavobacterium Johnsoniae. J. Ginseng Res. 36, 418–424. 10.5142/jgr.2012.36.4.418 23717145 PMC3659600

[B16] Hou J. Xue J. Lee M. Sung C. (2017). Ginsenoside Rd as a Potential Neuroprotective Agent Prevents Trimethyltin Injury. Biomed. Rep. 6, 435–440. 10.3892/br.2017.864 28413642 PMC5374896

[B17] Hu G. Wu Z. Yang F. Zhao H. Liu X. Deng Y. (2013). Ginsenoside Rd Blocks AIF Mitochondrio-Nuclear Translocation and NF-Κb Nuclear Accumulation by Inhibiting poly(ADP-Ribose) Polymerase-1 after Focal Cerebral Ischemia in Rats. Neurol. Sci. 34, 2101–2106. 10.1007/s10072-013-1344-6 23463404

[B18] Jeong H. G. Pokharel Y. R. Han E. H. Kang K. W. (2007). Induction of Cyclooxygenase-2 by Ginsenoside Rd via Activation of CCAAT-Enhancer Binding Proteins and Cyclic AMP Response Binding Protein. Biochem. Biophys. Res. Commun. 359, 51–56. 10.1016/j.bbrc.2007.05.034 17524357

[B19] Jin B. Zhang C. Geng Y. Liu M. (2020). Therapeutic Effect of Ginsenoside Rd on Experimental Autoimmune Encephalomyelitis Model Mice: Regulation of Inflammation and Treg/Th17 Cell Balance. Mediat. Inflamm. 2020, 8827527. 10.1155/2020/8827527 PMC7762661 33380901

[B20] Jin W. Ma R. Zhai L. Xu X. Lou T. Huang Q. (2020). Ginsenoside Rd Attenuates ACTH-Induced Corticosterone Secretion by Blocking the MC2R-cAMP/PKA/CREB Pathway in Y1 Mouse Adrenocortical Cells. Life Sci. 245, 117337. 10.1016/j.lfs.2020.117337 31972205

[B21] Jung E. Pyo M. K. Kim J. (2021). Pectin-Lyase-Modified Ginseng Extract and Ginsenoside Rd Inhibits High Glucose-Induced ROS Production in Mesangial Cells and Prevents Renal Dysfunction in Db/db Mice. Molecules 26. 10.3390/molecules26020367 PMC7828230 33445772

[B22] Jung S. C. Kim W. Park S. C. Jeong J. Park M. K. Lim S. (2014). Two Ginseng UDP-Glycosyltransferases Synthesize Ginsenoside Rg3 and Rd. Plant Cell Physiol. 55, 2177–2188. 10.1093/pcp/pcu147 25320211

[B23] Kang N. Gao H. He L. Liu Y. Fan H. Xu Q. (2021). Ginsenoside Rb1 Is an Immune-Stimulatory Agent with Antiviral Activity against Enterovirus 71. J. Ethnopharmacol. 266, 113401. 10.1016/j.jep.2020.113401 32980486

[B24] Kaviani M. Keshtkar S. Azarpira N. Hossein Aghdaei M. Geramizadeh B. Karimi M. H. (2019). Cytoprotective Effects of Ginsenoside Rd on Apoptosis-Associated Cell Death in the Isolated Human Pancreatic Islets. EXCLI J. 18, 666–676. 10.17179/excli2019-1698 31611749 PMC6785759

[B25] Kim B. J. (2013). Involvement of Melastatin Type Transient Receptor Potential 7 Channels in Ginsenoside Rd-Induced Apoptosis in Gastric and Breast Cancer Cells. J. Ginseng Res. 37, 201–209. 10.5142/jgr.2013.37.201 23717173 PMC3659640

[B26] Kim D. H. Chung J. H. Yoon J. S. Ha Y. M. Bae S. Lee E. K. (2013). Ginsenoside Rd Inhibits the Expressions of iNOS and COX-2 by Suppressing NF-Κb in LPS-Stimulated RAW264.7 Cells and Mouse Liver. J. Ginseng Res. 37, 54–63. 10.5142/jgr.2013.37.54 23717157 PMC3659628

[B27] Kim D. Y. Park Y. G. Quan H. Y. Kim S. J. Jung M. S. Chung S. H. (2012). Ginsenoside Rd Stimulates the Differentiation and Mineralization of Osteoblastic MC3T3-E1 Cells by Activating AMP-Activated Protein Kinase via the BMP-2 Signaling Pathway. Fitoterapia 83, 215–222. 10.1016/j.fitote.2011.10.017 22061660

[B28] Kim H. Kim J. H. Lee P. Y. Bae K. H. Cho S. Park B. C. (2013). Ginsenoside Rb1 Is Transformed into Rd and Rh2 by Microbacterium Trichothecenolyticum. J. Microbiol. Biotechnol. 23, 1802–1805. 10.4014/jmb.1307.07049 24018971

[B29] Kim H. I. Kim J. K. Kim J. Y. Han M. J. Kim D. H. (2019). Fermented Red Ginseng and Ginsenoside Rd Alleviate Ovalbumin-Induced Allergic Rhinitis in Mice by Suppressing IgE, Interleukin-4, and Interleukin-5 Expression. J. Ginseng Res. 43, 635–644. 10.1016/j.jgr.2019.02.006 31695569 PMC6823749

[B30] Kim J. H. Oh J. M. Chun S. Park H. Y. Im W. T. (2020). Enzymatic Biotransformation of Ginsenoside Rb2 into Rd by Recombinant α-L-Arabinopyranosidase from Blastococcus Saxobsidens. J. Microbiol. Biotechnol. 30, 391–397. 10.4014/jmb.1910.10065 31893597 PMC9728169

[B31] Kim K. A. Yoo H. H. Gu W. Yu D. H. Jin M. J. Choi H. L. (2014). Effect of a Soluble Prebiotic Fiber, NUTRIOSE, on the Absorption of Ginsenoside Rd in Rats Orally Administered Ginseng. J. Ginseng Res. 38, 203–207. 10.1016/j.jgr.2014.03.003 25378995 PMC4213839

[B32] Kim M. S. Yu J. M. Kim H. J. Kim H. B. Kim S. T. Jang S. K. (2014). Ginsenoside Re and Rd Enhance the Expression of Cholinergic Markers and Neuronal Differentiation in Neuro-2a Cells. Biol. Pharm. Bull. 37, 826–833. 10.1248/bpb.b14-00011 24599032

[B33] Kim N. D. Pokharel Y. R. Kang K. W. (2007). Ginsenoside Rd Enhances Glutathione Levels in H4IIE Cells via NF-kappaB-dependent Gamma-Glutamylcysteine Ligase Induction. Pharmazie 62, 933–936. 18214346

[B34] Kim W. K. Song S. Y. Oh W. K. Kaewsuwan S. Tran T. L. Kim W. S. (2013). Wound-healing Effect of Ginsenoside Rd from Leaves of Panax Ginseng via Cyclic AMP-dependent Protein Kinase Pathway. Eur. J. Pharmacol. 702, 285–293. 10.1016/j.ejphar.2013.01.048 23399764

[B35] Kim Y. J. Yamabe N. Choi P. Lee J. W. Ham J. Kang K. S. (2013). Efficient Thermal Deglycosylation of Ginsenoside Rd and its Contribution to the Improved Anticancer Activity of Ginseng. J. Agric. Food Chem. 61, 9185–9191. 10.1021/jf402774d 23984628

[B36] Li J. Xie Z. Z. Tang Y. B. Zhou J. G. Guan Y. Y. (2011). Ginsenoside-Rd, a Purified Component from Panax Notoginseng Saponins, Prevents Atherosclerosis in apoE Knockout Mice. Eur. J. Pharmacol. 652, 104–110. 10.1016/j.ejphar.2010.11.017 21122802

[B37] Li L. Li T. Tian X. Zhao L. (2021). Ginsenoside Rd Attenuates Tau Phosphorylation in Olfactory Bulb, Spinal Cord, and Telencephalon by Regulating Glycogen Synthase Kinase 3β and Cyclin-dependent Kinase 5. Evid. Based Complement. Altern. Med. 2021, 4485957. 10.1155/2021/4485957 PMC8720614 34987593

[B38] Li L. Liu J. Yan X. Qin K. Shi M. Lin T. (2011). Protective Effects of Ginsenoside Rd against Okadaic Acid-Induced Neurotoxicity *In Vivo* and *In Vitro* . J. Ethnopharmacol. 138, 135–141. 10.1016/j.jep.2011.08.068 21945003

[B39] Li L. Liu Z. Liu J. Tai X. Hu X. Liu X. (2013). Ginsenoside Rd Attenuates Beta-Amyloid-Induced Tau Phosphorylation by Altering the Functional Balance of Glycogen Synthase Kinase 3beta and Protein Phosphatase 2A. Neurobiol. Dis. 54, 320–328. 10.1016/j.nbd.2013.01.002 23321003

[B40] Li S. Y. Wang X. G. Ma M. M. Liu Y. Du Y. H. Lv X. F. (2012). Ginsenoside-Rd Potentiates Apoptosis Induced by Hydrogen Peroxide in Basilar Artery Smooth Muscle Cells through the Mitochondrial Pathway. Apoptosis 17, 113–120. 10.1007/s10495-011-0671-4 22076303

[B41] Li X. Y. Liang J. Tang Y. B. Zhou J. G. Guan Y. Y. (2010). Ginsenoside Rd Prevents Glutamate-Induced Apoptosis in Rat Cortical Neurons. Clin. Exp. Pharmacol. Physiol. 37, 199–204. 10.1111/j.1440-1681.2009.05286.x 19719747

[B42] Lin F. Guo X. Lu W. (2015). Efficient Biotransformation of Ginsenoside Rb1 to Rd by Isolated Aspergillus versicolor, Excreting β-glucosidase in the Spore Production Phase of Solid Culture. Ant. Van Leeuwenhoek 108, 1117–1127. 10.1007/s10482-015-0565-5 26373416

[B43] Lin T. Liu Y. Shi M. Liu X. Li L. Liu Y. (2012). Promotive Effect of Ginsenoside Rd on Proliferation of Neural Stem Cells *In Vivo* and *In Vitro* . J. Ethnopharmacol. 142, 754–761. 10.1016/j.jep.2012.05.057 22683911

[B44] Liu C. Wang J. Yang Y. Liu X. Zhu Y. Zou J. (2018). Ginsenoside Rd Ameliorates Colitis by Inducing P62-Driven Mitophagy-Mediated NLRP3 Inflammasome Inactivation in Mice. Biochem. Pharmacol. 155, 366–379. 10.1016/j.bcp.2018.07.010 30012462

[B45] Liu G. M. Lu T. C. Sun M. L. Jia W. Y. Ji X. Luo Y. G. (2020). Ginsenoside Rd Inhibits Glioblastoma Cell Proliferation by Up-Regulating the Expression of miR-144-5p. Biol. Pharm. Bull. 43, 1534–1541. 10.1248/bpb.b20-00338 32999164

[B46] Liu H. Lu X. Hu Y. Fan X. (2020). Chemical Constituents of Panax Ginseng and Panax Notoginseng Explain Why They Differ in Therapeutic Efficacy. Pharmacol. Res. 161, 105263. 10.1016/j.phrs.2020.105263 33127555

[B47] Liu J. Yan X. Li L. Li Y. Zhou L. Zhang X. (2015). Ginsenoside Rd Improves Learning and Memory Ability in APP Transgenic Mice. J. Mol. Neurosci. 57, 522–528. 10.1007/s12031-015-0632-4 26358038

[B48] Liu J. F. Yan X. D. Qi L. S. Li L. Hu G. Y. Li P. (2015). Ginsenoside Rd Attenuates Aβ25-35-Induced Oxidative Stress and Apoptosis in Primary Cultured Hippocampal Neurons. Chem. Biol. Interact. 239, 12–18. 10.1016/j.cbi.2015.06.030 26111763

[B49] Liu Q. M. Jung H. M. Cui C. H. Sung B. H. Kim J. K. Kim S. G. (2013). Bioconversion of Ginsenoside Rc into Rd by a Novel α-L-arabinofuranosidase, Abf22-3 from Leuconostoc Sp. 22-3: Cloning, Expression, and Enzyme Characterization. Ant. Van Leeuwenhoek 103, 747–754. 10.1007/s10482-012-9856-2 23224374

[B50] Liu X. Wang L. Wen A. Yang J. Yan Y. Song Y. (2012). Ginsenoside-Rd Improves Outcome of Acute Ischaemic Stroke - a Randomized, Double-Blind, Placebo-Controlled, Multicenter Trial. Eur. J. Neurol. 19, 855–863. 10.1111/j.1468-1331.2011.03634.x 22233205

[B51] Liu X. Xia J. Wang L. Song Y. Yang J. Yan Y. (2009). Efficacy and Safety of Ginsenoside-Rd for Acute Ischaemic Stroke: a Randomized, Double-Blind, Placebo-Controlled, Phase II Multicenter Trial. Eur. J. Neurol. 16, 569–575. 10.1111/j.1468-1331.2009.02534.x 19236467

[B52] Liu X. Y. Zhou X. Y. Hou J. C. Zhu H. Wang Z. Liu J. X. (2015). Ginsenoside Rd Promotes Neurogenesis in Rat Brain after Transient Focal Cerebral Ischemia via Activation of PI3K/Akt Pathway. Acta Pharmacol. Sin. 36, 421–428. 10.1038/aps.2014.156 25832422 PMC4387301

[B53] Liu Y. Zhang R. Y. Zhao J. Dong Z. Feng D. Y. Wu R. (2015). Ginsenoside Rd Protects SH-Sy5y Cells against 1-Methyl-4-Phenylpyridinium Induced Injury. Int. J. Mol. Sci. 16, 14395–14408. 10.3390/ijms160714395 26114390 PMC4519848

[B54] Park C. S. Yoo M. H. Noh K. H. Oh D. K. (2010). Biotransformation of Ginsenosides by Hydrolyzing the Sugar Moieties of Ginsenosides Using Microbial Glycosidases. Appl. Microbiol. Biotechnol. 87, 9–19. 10.1007/s00253-010-2567-6 20376631

[B55] Phi L. T. H. Sari I. N. Wijaya Y. T. Kim K. S. Park K. Cho A. E. (2019). Ginsenoside Rd Inhibits the Metastasis of Colorectal Cancer via Epidermal Growth Factor Receptor Signaling Axis. IUBMB Life 71, 601–610. 10.1002/iub.1984 30576064

[B56] Pokharel Y. R. Kim N. D. Han H. K. Oh W. K. Kang K. W. (2010). Increased Ubiquitination of Multidrug Resistance 1 by Ginsenoside Rd. Nutr. Cancer 62, 252–259. 10.1080/01635580903407171 20099200

[B57] Quan L. H. Piao J. Y. Min J. W. Kim H. B. Kim S. R. Yang D. U. (2011). Biotransformation of Ginsenoside Rb1 to Prosapogenins, Gypenoside XVII, Ginsenoside Rd, Ginsenoside F2, and Compound K by Leuconostoc Mesenteroides DC102. J. Ginseng Res. 35, 344–351. 10.5142/jgr.2011.35.3.344 23717079 PMC3659545

[B58] Ren K. Jin C. Ma P. Ren Q. Jia Z. Zhu D. (2016). Ginsenoside Rd Alleviates Mouse Acute Renal Ischemia/reperfusion Injury by Modulating Macrophage Phenotype. J. Ginseng Res. 40, 196–202. 10.1016/j.jgr.2015.12.003 27158241 PMC4845042

[B59] Ren K. Li S. Ding J. Zhao S. Liang S. Cao X. (2021). Ginsenoside Rd Attenuates Mouse Experimental Autoimmune Neuritis by Modulating Monocyte Subsets Conversion. Biomed. Pharmacother. 138, 111489. 10.1016/j.biopha.2021.111489 33743332

[B60] Renchinkhand G. Cho S. H. Park Y. W. Song G. Y. Nam M. S. (2020). Biotransformation of Major Ginsenoside Rb1 to Rd by Dekkera Anomala YAE-1 from Mongolian Fermented Milk (Airag). J. Microbiol. Biotechnol. 30, 1536–1542. 10.4014/jmb.2004.04022 32807763 PMC9728303

[B61] Renchinkhand G. Cho S. H. Urgamal M. Park Y. W. Nam J. H. Bae H. C. (2017). Characterization of Paenibacillus Sp. MBT213 Isolated from Raw Milk and its Ability to Convert Ginsenoside Rb1 into Ginsenoside Rd from Panax Ginseng. Korean J. Food Sci. Anim. Resour. 37, 735–742. 10.5851/kosfa.2017.37.5.735 29147097 PMC5686332

[B62] Sarhene M. Ni J. Y. Duncan E. S. Liu Z. Li S. Zhang J. (2021). Ginsenosides for Cardiovascular Diseases; Update on Pre-clinical and Clinical Evidence, Pharmacological Effects and the Mechanisms of Action. Pharmacol. Res. 166, 105481. 10.1016/j.phrs.2021.105481 33549726

[B63] Shi Q. Hao Q. Bouissac J. Lu Y. Tian S. Luu B. (2005). Ginsenoside-Rd from Panax Notoginseng Enhances Astrocyte Differentiation from Neural Stem Cells. Life Sci. 76, 983–995. 10.1016/j.lfs.2004.07.026 15607328

[B64] Shin K. C. Lee G. W. Oh D. K. (2013). Production of Ginsenoside Rd from Ginsenoside Rc by α-L-arabinofuranosidase from Caldicellulosiruptor Saccharolyticus. J. Microbiol. Biotechnol. 23, 483–488. 10.4014/jmb.1211.11012 23568202

[B65] Shin K. C. Oh D. K. (2016). Classification of Glycosidases that Hydrolyze the Specific Positions and Types of Sugar Moieties in Ginsenosides. Crit. Rev. Biotechnol. 36, 1036–1049. 10.3109/07388551.2015.1083942 26383974

[B66] Son J. W. Kim H. J. Oh D. K. (2008). Ginsenoside Rd Production from the Major Ginsenoside Rb(1) by Beta-Glucosidase from Thermus Caldophilus. Biotechnol. Lett. 30, 713–716. 10.1007/s10529-007-9590-4 17989924

[B67] Sun D. Wang B. Shi M. Zhang Y. X. Zhou L. F. Liu Z. R. (2012). Pharmacokinetic, Tissue Distribution and Excretion of Ginsenoside-Rd in Rodents. Phytomedicine 19, 369–373. 10.1016/j.phymed.2011.08.061 21899993

[B68] Tamura T. Cui X. Sakaguchi N. Akashi M. (2008). Ginsenoside Rd Prevents and Rescues Rat Intestinal Epithelial Cells from Irradiation-Induced Apoptosis. Food Chem. Toxicol. 46, 3080–3089. 10.1016/j.fct.2008.06.011 18638517

[B69] Tian Y. Z. Liu Y. P. Tian S. C. Ge S. Y. Wu Y. J. Zhang B. L. (2020). Antitumor Activity of Ginsenoside Rd in Gastric Cancer via Up-Regulation of Caspase-3 and Caspase-9. Pharmazie 75, 147–150. 10.1691/ph.2020.9931 32295691

[B70] Wang B. Feng G. Tang C. Wang L. Cheng H. Zhang Y. (2013). Ginsenoside Rd Maintains Adult Neural Stem Cell Proliferation during Lead-Impaired Neurogenesis. Neurol. Sci. 34, 1181–1188. 10.1007/s10072-012-1215-6 23073826

[B71] Wang B. Zhu Q. Man X. Guo L. Hao L. (2014). Ginsenoside Rd Inhibits Apoptosis Following Spinal Cord Ischemia/reperfusion Injury. Neural Regen. Res. 9, 1678–1687. 10.4103/1673-5374.141802 25374589 PMC4211188

[B72] Wang F. Park J. S. Ma Y. Ma H. Lee Y. J. Lee G. R. (2021). Ginseng Saponin Enriched in Rh1 and Rg2 Ameliorates Nonalcoholic Fatty Liver Disease by Inhibiting Inflammasome Activation. Nutrients 13. 10.3390/nu13030856 PMC7999915 33807927

[B73] Wang H. Jiang N. Lv J. Huang H. Liu X. (2020). Ginsenoside Rd Reverses Cognitive Deficits by Modulating BDNF-dependent CREB Pathway in Chronic Restraint Stress Mice. Life Sci. 258, 118107. 10.1016/j.lfs.2020.118107 32682919

[B74] Wang L. Zhang Y. Chen J. Li S. Wang Y. Hu L. (2012). Immunosuppressive Effects of Ginsenoside-Rd on Skin Allograft Rejection in Rats. J. Surg. Res. 176, 267–274. 10.1016/j.jss.2011.06.038 21872268

[B75] Wang L. Zhang Y. Wang Z. Li S. Min G. Wang L. (2012). Inhibitory Effect of Ginsenoside-Rd on Carrageenan-Induced Inflammation in Rats. Can. J. Physiol. Pharmacol. 90, 229–236. 10.1139/y11-127 22300288

[B76] Wang P. Du X. Xiong M. Cui J. Yang Q. Wang W. (2016). Ginsenoside Rd Attenuates Breast Cancer Metastasis Implicating Derepressing microRNA-18a-Regulated Smad2 Expression. Sci. Rep. 6, 33709. 10.1038/srep33709 27641158 PMC5027393

[B77] Wang Q. Fu W. Yu X. Xu H. Sui D. Wang Y. (2021). Ginsenoside Rg2 Alleviates Myocardial Fibrosis by Regulating TGF-β1/Smad Signalling Pathway. Pharm. Biol. 59, 106–113. 10.1080/13880209.2020.1867197 33535854 PMC8871615

[B78] Wang W. Wang G. J. Xie H. T. Sun J. G. Zhao S. Jiang X. L. (2007). Determination of Ginsenoside Rd in Dog Plasma by Liquid Chromatography-Mass Spectrometry after Solid-phase Extraction and its Application in Dog Pharmacokinetics Studies. J. Chromatogr. B Anal. Technol. Biomed. Life Sci. 852, 8–14. 10.1016/j.jchromb.2006.12.046 17267298

[B79] Wang Y. Li X. Wang X. Lau W. Wang Y. Xing Y. (2013). Ginsenoside Rd Attenuates Myocardial Ischemia/reperfusion Injury via Akt/GSK-3β Signaling and Inhibition of the Mitochondria-dependent Apoptotic Pathway. PLoS One 8, e70956. 10.1371/journal.pone.0070956 23976968 PMC3745454

[B80] Wu S. D. Xia F. Lin X. M. Duan K. L. Wang F. Lu Q. L. (2016). Ginsenoside-Rd Promotes Neurite Outgrowth of PC12 Cells through MAPK/ERK- and PI3K/AKT-dependent Pathways. Int. J. Mol. Sci. 17. 10.3390/ijms17020177 PMC4783911 26840295

[B81] Xie J. Zhao D. Zhao L. Pei J. Xiao W. Ding G. (2016). Characterization of a Novel Arabinose-Tolerant α-L-arabinofuranosidase with High Ginsenoside Rc to Ginsenoside Rd Bioconversion Productivity. J. Appl. Microbiol. 120, 647–660. 10.1111/jam.13040 26725313

[B82] Xie Z. Shi M. Zhang C. Zhao H. Hui H. Zhao G. (2016). Ginsenoside Rd Protects against Cerebral Ischemia-Reperfusion Injury via Decreasing the Expression of the NMDA Receptor 2B Subunit and its Phosphorylated Product. Neurochem. Res. 41, 2149–2159. 10.1007/s11064-016-1930-0 27165636

[B83] Xu X. F. Qu W. J. Jia Z. Han T. Liu M. N. Bai Y. Y. (2021). Effect of Cultivation Ages on Anti-inflammatory Activity of a New Type of Red Ginseng. Biomed. Pharmacother. 136, 111280. 10.1016/j.biopha.2021.111280 33485063

[B84] Yan X. Hu G. Yan W. Chen T. Yang F. Zhang X. (2017). Ginsenoside Rd Promotes Non-amyloidogenic Pathway of Amyloid Precursor Protein Processing by Regulating Phosphorylation of Estrogen Receptor Alpha. Life Sci. 168, 16–23. 10.1016/j.lfs.2016.11.002 27825720

[B85] Yang B. Wang R. Ji L. L. Li X. P. Li X. H. Zhou H. G. (2021). Exploration of the Function of Ginsenoside RD Attenuates Lipopolysaccharide-Induced Lung Injury: A Study of Network Pharmacology and Experimental Validation. Augusta, Ga: Shock. 10.1097/SHK.0000000000001824 34172615

[B86] Yang L. Deng Y. Xu S. Zeng X. (2007). *In Vivo* pharmacokinetic and Metabolism Studies of Ginsenoside Rd. J. Chromatogr. B Anal. Technol. Biomed. Life Sci. 854, 77–84. 10.1016/j.jchromb.2007.04.014 17526438

[B87] Yang L. Xu S. J. Zeng X. Liu Y. M. Deng S. G. Wu Z. F. (2006). Determination of Ginsenoside Rd and its Metabolites in Rat Urine by LC-MS. Yao Xue Xue Bao 41, 742–746. 17039780

[B88] Yang L. X. Zhang X. Zhao G. (2016). Ginsenoside Rd Attenuates DNA Damage by Increasing Expression of DNA Glycosylase Endonuclease VIII-like Proteins after Focal Cerebral Ischemia. Chin. Med. J. Engl. 129, 1955–1962. 10.4103/0366-6999.187851 27503022 PMC4989428

[B89] Yang X. Gao M. Miao M. Jiang C. Zhang D. Yin Z. (2021). Combining Combretastatin A4 Phosphate with Ginsenoside Rd Synergistically Inhibited Hepatocellular Carcinoma by Reducing HIF-1α via PI3K/AKT/mTOR Signalling Pathway. J. Pharm. Pharmacol. 73, 263–271. 10.1093/jpp/rgaa006 33793802 PMC8531789

[B90] Yang X. L. Guo T. K. Wang Y. H. Gao M. T. Qin H. Wu Y. J. (2012). Therapeutic Effect of Ginsenoside Rd in Rats with TNBS-Induced Recurrent Ulcerative Colitis. Arch. Pharm. Res. 35, 1231–1239. 10.1007/s12272-012-0714-6 22864746

[B91] Yang X. L. Guo T. K. Wang Y. H. Huang Y. H. Liu X. Wang X. X. (2012). Ginsenoside Rd Attenuates the Inflammatory Response via Modulating P38 and JNK Signaling Pathways in Rats with TNBS-Induced Relapsing Colitis. Int. Immunopharmacol. 12, 408–414. 10.1016/j.intimp.2011.12.014 22227208

[B92] Yang Z. Chen A. Sun H. Ye Y. Fang W. (2007). Ginsenoside Rd Elicits Th1 and Th2 Immune Responses to Ovalbumin in Mice. Vaccine 25, 161–169. 10.1016/j.vaccine.2006.05.075 16950547

[B93] Yang Z. G. Sun H. X. Ye Y. P. (2006). Ginsenoside Rd from Panax Notoginseng Is Cytotoxic towards HeLa Cancer Cells and Induces Apoptosis. Chem. Biodivers. 3, 187–197. 10.1002/cbdv.200690022 17193257

[B94] Yao L. Han Z. Zhao G. Xiao Y. Zhou X. Dai R. (2020). Ginsenoside Rd Ameliorates High Fat Diet-Induced Obesity by Enhancing Adaptive Thermogenesis in a cAMP-dependent Manner. Obes. (Silver Spring) 28, 783–792. 10.1002/oby.22761 32144882

[B95] Ye L. Zhang C. Li J. Shi X. Feng M. (2012). Effects of External Calcium on the Biotransformation of Ginsenoside Rb1 to Ginsenoside Rd by Paecilomyces Bainier 229-7. World J. Microbiol. Biotechnol. 28, 857–863. 10.1007/s11274-011-0882-4 22805805

[B96] Ye L. Zhou C. Q. Zhou W. Zhou P. Chen D. F. Liu X. H. (2010). Biotransformation of Ginsenoside Rb1 to Ginsenoside Rd by Highly Substrate-Tolerant Paecilomyces Bainier 229-7. Bioresour. Technol. 101, 7872–7876. 10.1016/j.biortech.2010.04.102 20605716

[B97] Ye R. Han J. Kong X. Zhao L. Cao R. Rao Z. (2008). Protective Effects of Ginsenoside Rd on PC12 Cells against Hydrogen Peroxide. Biol. Pharm. Bull. 31, 1923–1927. 10.1248/bpb.31.1923 18827356

[B98] Ye R. Kong X. Yang Q. Zhang Y. Han J. Li P. (2011). Ginsenoside Rd in Experimental Stroke: Superior Neuroprotective Efficacy with a Wide Therapeutic Window. Neurotherapeutics 8, 515–525. 10.1007/s13311-011-0051-3 21647765 PMC3250281

[B99] Ye R. Kong X. Yang Q. Zhang Y. Han J. Zhao G. (2011). Ginsenoside Rd Attenuates Redox Imbalance and Improves Stroke Outcome after Focal Cerebral Ischemia in Aged Mice. Neuropharmacology 61, 815–824. 10.1016/j.neuropharm.2011.05.029 21664366

[B100] Ye R. Li N. Han J. Kong X. Cao R. Rao Z. (2009). Neuroprotective Effects of Ginsenoside Rd against Oxygen-Glucose Deprivation in Cultured Hippocampal Neurons. Neurosci. Res. 64, 306–310. 10.1016/j.neures.2009.03.016 19447300

[B101] Ye R. Yang Q. Kong X. Han J. Zhang X. Zhang Y. (2011). Ginsenoside Rd Attenuates Early Oxidative Damage and Sequential Inflammatory Response after Transient Focal Ischemia in Rats. Neurochem. Int. 58, 391–398. 10.1016/j.neuint.2010.12.015 21185898

[B102] Ye R. Zhang X. Kong X. Han J. Yang Q. Zhang Y. (2011). Ginsenoside Rd Attenuates Mitochondrial Dysfunction and Sequential Apoptosis after Transient Focal Ischemia. Neuroscience 178, 169–180. 10.1016/j.neuroscience.2011.01.007 21219973

[B103] Yi Y. S. (2021). New Mechanisms of Ginseng Saponin-Mediated Anti-inflammatory Action via Targeting Canonical Inflammasome Signaling Pathways. J. Ethnopharmacol. 278, 114292. 10.1016/j.jep.2021.114292 34089812

[B104] Yokozawa T. Dong E. (2001). Role of Ginsenoside-Rd in Cisplatin-Induced Renal Injury: Special Reference to DNA Fragmentation. Nephron 89, 433–438. 10.1159/000046116 11721162

[B105] Yokozawa T. Liu Z. W. Dong E. (1998). A Study of Ginsenoside-Rd in a Renal Ischemia-Reperfusion Model. Nephron 78, 201–206. 10.1159/000044911 9496738

[B106] Yokozawa T. Liu Z. W. (2000). The Role of Ginsenoside-Rd in Cisplatin-Induced Acute Renal Failure. Ren. Fail 22, 115–127. 10.1081/jdi-100100858 10803758

[B107] Yokozawa T. Owada S. (1999). Effect of Ginsenoside-Rd in Cephaloridine-Induced Renal Disorder. Nephron 81, 200–207. 10.1159/000045277 9933756

[B108] Yokozawa T. Satoh A. Cho E. J. (2004). Ginsenoside-Rd Attenuates Oxidative Damage Related to Aging in Senescence-Accelerated Mice. J. Pharm. Pharmacol. 56, 107–113. 10.1211/0022357022449 14980007

[B109] Yoon J. H. Choi Y. J. Cha S. W. Lee S. G. (2012). Anti-metastatic Effects of Ginsenoside Rd via Inactivation of MAPK Signaling and Induction of Focal Adhesion Formation. Phytomedicine 19, 284–292. 10.1016/j.phymed.2011.08.069 21982435

[B110] Yu X. Li H. Lin D. Guo W. Xu Z. Wang L. (2021). Ginsenoside Prolongs the Lifespan of *C. elegans* via Lipid Metabolism and Activating the Stress Response Signaling Pathway. Int. J. Mol. Sci. 22. 10.3390/ijms22189668 PMC8465798 34575832

[B111] Zeng X. Deng Y. Feng Y. Liu Y. Yang L. Huang Y. (2010). Pharmacokinetics and Safety of Ginsenoside Rd Following a Single or Multiple Intravenous Dose in Healthy Chinese Volunteers. J. Clin. Pharmacol. 50, 285–292. 10.1177/0091270009344334 19940231

[B112] Zhang B. Hu X. Wang H. Wang R. Sun Z. Tan X. (2020). Effects of a Dammarane-type Saponin, Ginsenoside Rd, in Nicotine-Induced Vascular Endothelial Injury. Phytomedicine 79, 153325. 10.1016/j.phymed.2020.153325 32920289

[B113] Zhang C. Du F. Shi M. Ye R. Cheng H. Han J. (2012). Ginsenoside Rd Protects Neurons against Glutamate-Induced Excitotoxicity by Inhibiting Ca(2+) Influx. Cell Mol. Neurobiol. 32, 121–128. 10.1007/s10571-011-9742-x 21811848 PMC11498599

[B114] Zhang C. Liu X. Xu H. Hu G. Zhang X. Xie Z. (2020). Protopanaxadiol Ginsenoside Rd Protects against NMDA Receptor-Mediated Excitotoxicity by Attenuating Calcineurin-Regulated DAPK1 Activity. Sci. Rep. 10, 8078. 10.1038/s41598-020-64738-2 32415270 PMC7228936

[B115] Zhang E. Shi H. Yang L. Wu X. Wang Z. (2017). Ginsenoside Rd Regulates the Akt/mTOR/p70S6K Signaling Cascade and Suppresses Angiogenesis and Breast Tumor Growth. Oncol. Rep. 38, 359–367. 10.3892/or.2017.5652 28534996

[B116] Zhang G. Xia F. Zhang Y. Zhang X. Cao Y. Wang L. (2016). Ginsenoside Rd Is Efficacious against Acute Ischemic Stroke by Suppressing Microglial Proteasome-Mediated Inflammation. Mol. Neurobiol. 53, 2529–2540. 10.1007/s12035-015-9261-8 26081140

[B117] Zhang H. Park S. Huang H. Kim E. Yi J. Choi S. K. (2021). Anticancer Effects and Potential Mechanisms of Ginsenoside Rh2 in Various Cancer Types (Review). Oncol. Rep. 45. 10.3892/or.2021.7984 33649861

[B118] Zhang R. Tan S. Q. Zhang B. L. Guo Z. Y. Tian L. Y. Weng P. (2021). Two Key Amino Acids Variant of Alpha-L-Arabinofuranosidase from Bacillus Subtilis Str. 168 with Altered Activity for Selective Conversion Ginsenoside Rc to Rd. Molecules 26, 1733. 10.3390/molecules26061733 33808840 PMC8003784

[B119] Zhang X. Liu X. Hu G. Zhang G. Zhao G. Shi M. (2020). Ginsenoside Rd Attenuates Blood-Brain Barrier Damage by Suppressing Proteasome-Mediated Signaling after Transient Forebrain Ischemia. Neuroreport 31, 466–472. 10.1097/WNR.0000000000001426 32168101

[B120] Zhang X. Shi M. Bjørås M. Wang W. Zhang G. Han J. (2013). Ginsenoside Rd Promotes Glutamate Clearance by Up-Regulating Glial Glutamate Transporter GLT-1 via PI3K/AKT and ERK1/2 Pathways. Front. Pharmacol. 4, 152. 10.3389/fphar.2013.00152 24376419 PMC3858668

[B121] Zhang X. Shi M. Ye R. Wang W. Liu X. Zhang G. (2014). Ginsenoside Rd Attenuates Tau Protein Phosphorylation via the PI3K/AKT/GSK-3β Pathway after Transient Forebrain Ischemia. Neurochem. Res. 39, 1363–1373. 10.1007/s11064-014-1321-3 24792734

[B122] Zhang Y. Zhou L. Zhang X. Bai J. Shi M. Zhao G. (2012). Ginsenoside-Rd Attenuates TRPM7 and ASIC1a but Promotes ASIC2a Expression in Rats after Focal Cerebral Ischemia. Neurol. Sci. 33, 1125–1131. 10.1007/s10072-011-0916-6 22231470

[B123] Zhang Y. X. Wang L. Xiao E. L. Li S. J. Chen J. J. Gao B. (2013). Ginsenoside-Rd Exhibits Anti-inflammatory Activities through Elevation of Antioxidant Enzyme Activities and Inhibition of JNK and ERK Activation *In Vivo* . Int. Immunopharmacol. 17, 1094–1100. 10.1016/j.intimp.2013.10.013 24455777

[B124] Zhao X. Wang J. Li J. Fu L. Gao J. Du X. (2009). Highly Selective Biotransformation of Ginsenoside Rb1 to Rd by the Phytopathogenic Fungus Cladosporium Fulvum (Syn. Fulvia Fulva). J. Ind. Microbiol. Biotechnol. 36, 721–726. 10.1007/s10295-009-0542-y 19229572

[B125] Zhong F. L. Ma R. Jiang M. Dong W. W. Jiang J. Wu S. (2016). Cloning and Characterization of Ginsenoside-Hydrolyzing β-Glucosidase from Lactobacillus Brevis that Transforms Ginsenosides Rb1 and F2 into Ginsenoside Rd and Compound K. J. Microbiol. Biotechnol. 26, 1661–1667. 10.4014/jmb.1605.05052 27435543

[B126] Zhou J. S. Wang J. F. He B. R. Cui Y. S. Fang X. Y. Ni J. L. (2014). Ginsenoside Rd Attenuates Mitochondrial Permeability Transition and Cytochrome C Release in Isolated Spinal Cord Mitochondria: Involvement of Kinase-Mediated Pathways. Int. J. Mol. Sci. 15, 9859–9877. 10.3390/ijms15069859 24897022 PMC4100126

[B127] Zhou S. Ji Y. Yao H. Guo H. Zhang Z. Wang Z. (2022). Application of Ginsenoside Rd in Periodontitis with Inhibitory Effects on Pathogenicity, Inflammation, and Bone Resorption. Front. Cell Infect. Microbiol. 12, 813953. 10.3389/fcimb.2022.813953 35480231 PMC9035930

[B128] Zhu D. Liu M. Yang Y. Ma L. Jiang Y. Zhou L. (2014). Ginsenoside Rd Ameliorates Experimental Autoimmune Encephalomyelitis in C57BL/6 Mice. J. Neurosci. Res. 92, 1217–1226. 10.1002/jnr.23397 24798871

